# Effective method of monitoring cerebral tissue oxygen saturation in cardiac surgery patients by combined use of tNIRS-1 and bispectral index

**DOI:** 10.1038/s41598-021-03527-x

**Published:** 2021-12-16

**Authors:** A. Sugiura, K. Torii, H. Tsutsumi, T. Someya, D. Yasuoka, K. Nishikiori, D. Kitahara, H. Kakinuma

**Affiliations:** Department of Clinical Engineering, Showa University Northern Yokohama Hospital, 35-1, Chigasaki-chuo, Tsuzuki ward, Yokohama city, Kanagawa 224-8503 Japan

**Keywords:** Biomedical engineering, Near-infrared spectroscopy

## Abstract

To continuously and noninvasively monitor the cerebral tissue oxygen saturation (StO_2_) and hemoglobin concentration (gasHb) in cardiac surgery patients, a method combining the use of a cerebral tissue oximeter using near infrared time-resolved spectroscopy (tNIRS-1) and the bispectral index (BIS) was developed in this study. Moreover, the correlation between the estimated hemoglobin concentration (estHb), measured via tNIRS-1, and the hemoglobin concentration (gasHb), analyzed using a blood gas analyzer, were compared. The relationship between the BIS and gasHb was also examined. Through the comparison of BIS and StO_2_ (r1), and estHb and gasHb (r2), the correlation between the two was clarified with maximum r1 and r2 values of 0.617 and 0.946, respectively. The relationship between BIS and gasHb (r3), showed that there was a favorable correlation with a maximum r3 value of 0.969. There was also a continuous correlation between BIS and StO_2_ in patients undergoing cardiac surgery. In addition, a strong correlation was found between estHb and gasHb, and between BIS and gasHb. It was therefore concluded that the combined use of BIS and tNIRS-1 is useful to evaluate cerebral hypoxia, allowing for quick response to cerebral hypoxia and reduction of hemoglobin concentration during the operation.

## Introduction

Cardiac surgery is a procedure highly invasive to the patient’s body, requiring monitoring of the patient’s physiological state as it changes continuously. In particular, understanding the state of the brain during surgery (cerebral tissue oxygen saturation, electroencephalogram (EEG), etc.) is indispensable from the perspective of brain protection to prevent irreversible damage to the brain. In addition, noninvasive monitoring methods should be used in consideration of patient safety.

Near infrared spectroscopy (NIRS) is one method used to monitor cerebral tissue oxygen saturation noninvasively; it was first reported in 1977 by Jöbsis at Duke University, who demonstrated the relationship between light attenuation and hemoglobin concentration in blood upon irradiation with light based on Beer–Lambert’s law^1^.

Various methods for monitoring cerebral tissue oxygen saturation using near infrared light, such as spatially resolved spectroscopy (SRS), phase resolved spectroscopy (PRS) and time resolved spectroscopy (TRS), have been reported^2–5^. Among them, TRS has been used to measure TRS-10 and TRS-20, which are laboratory instruments, and has been used in many fields.

The tNIRS-1 was the first instrument for which TRS was employed in clinical use^6^. The TRS method employed is highly quantitative and enables the continuous and noninvasive monitoring of the absolute values of cerebral tissue oxygen saturation and estimation of the hemoglobin concentration. In contrast, NIRS, which has been used in clinical devices, is used to obtain relative values that can be easily affected by head shape and slight deviations in the position of the attached sensors^7,8^. Therefore, its application has been limited in terms of quantitative analyses.

In this study, the absolute values of cerebral tissue oxygen saturation (StO_2_), obtained using tNIRS-1 for cardiac surgery patients, were continuously compared with the values obtained using the brain waves measuring instrument (BIS), which has been used as an indicator of brain ischemia as well as the depth and sedation of anesthesia.

The relationship between estimated hemoglobin concentration (estHb), measured by tNIRS-1, and the hemoglobin concentration (gasHb), measured by the blood gas analyzer, were also analyzed and compared. Moreover, we examined the correlation between cerebral hypoxia by comparing BIS with gasHb.

Based on these results, we confirmed that tNIRS-1 and BIS can be combined and used as an effective evaluation method to continuously, noninvasively, and accurately determined the oxygen state of brain tissue.

## Results

### Comparison of StO_2_ and BIS

The effectiveness of tNIRS-1 during cardiac surgery was evaluated by comparing the correlation between StO_2_ and estHb values measured by tNIRS-1, with BIS values measured by BIS and gasHb values measured by the blood gas analyzer.

**Figure 1:**
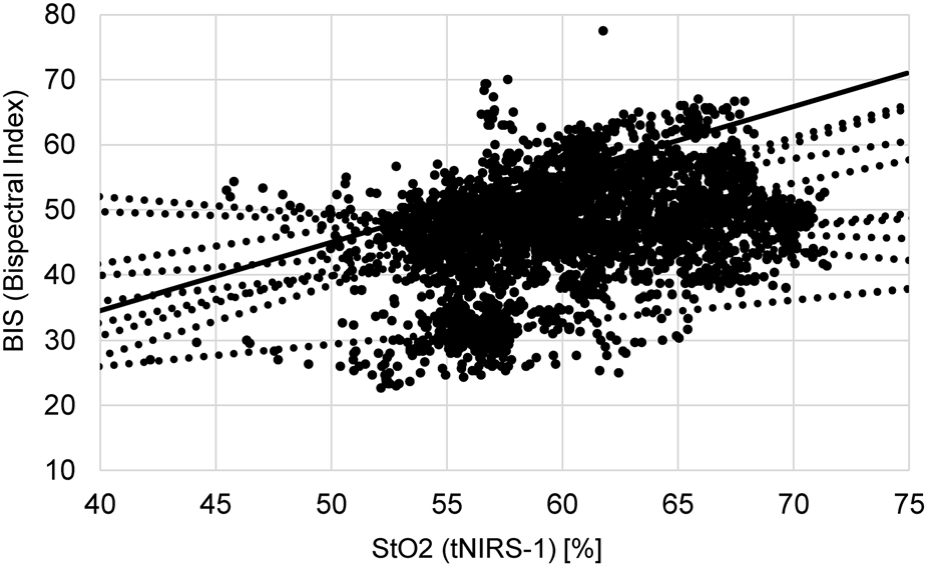
Correlation between StO_2_ and BIS. This graph compares the change of BIS with cerebral tissue oxygen saturation, measured noninvasively and continuously by sensors attached to patient foreheads.

Figure 1 shows the comparison of StO_2_ and BIS. Both parameters were measured continuously during the cardiac surgery. The comparison of StO_2_ and BIS showed a correlation with a maximum r1 value of 0.617, and hence confirmed that tNIRS-1 and BIS can be combined to monitor the cerebral oxygen saturation continuously, noninvasively, and effectively.

### Comparison of estHb and gasHb

**Figure 2:**
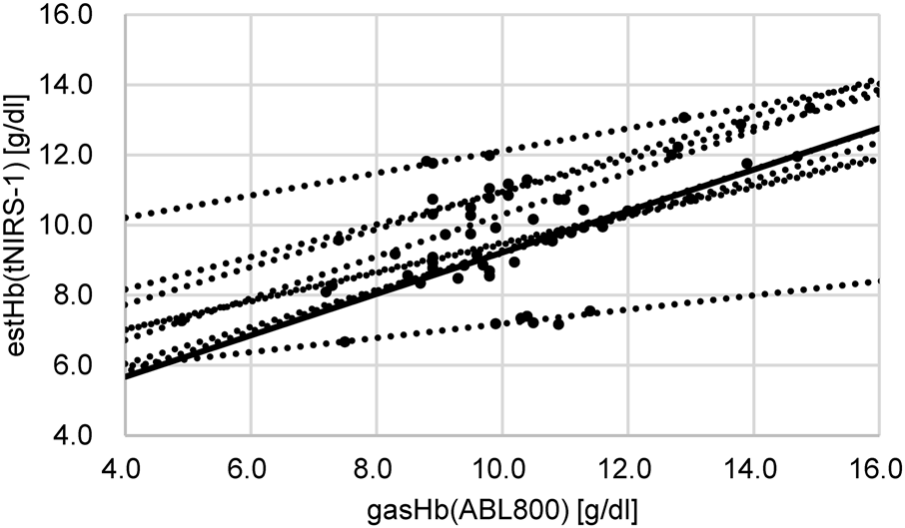
Correlation between gasHb and estHb. This graph compares the change of cerebral tissue oxygen saturation to the hemoglobin concentration measured every 30 min by a blood gas analyzer.

For the comparison of estHb and gasHb (Fig. 2), estHb was measured continuously, and gasHb was measured every 30 min. These measurement values showed a good correlation with the maximum r2 value of 0.946. As a result, it was confirmed that the value of estHb by tNIRS-1, which was used noninvasively and continuously, was reliable for determining the absolute hemoglobin concentration.

### Comparison of gasHb and BIS

**Figure 3:**
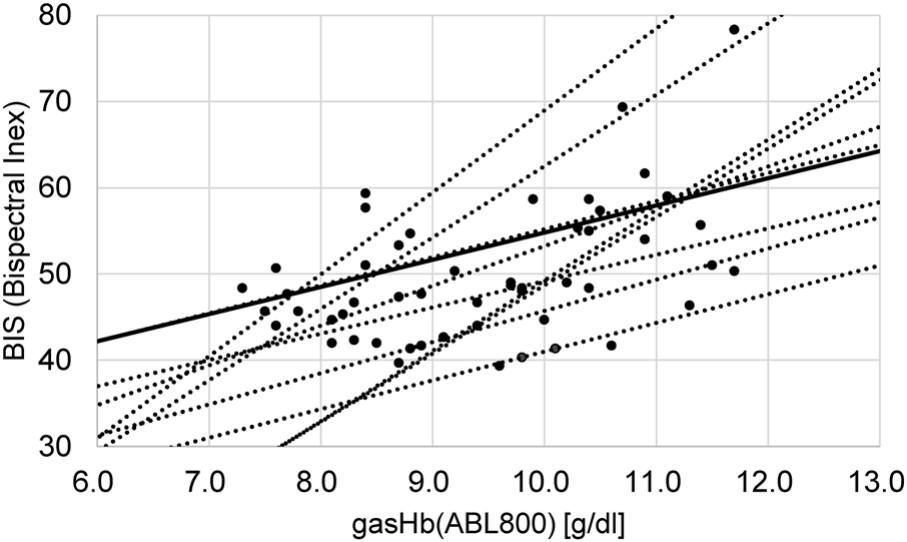
Correlation between gasHb and BIS. This is a comparison of the change in BIS to the hemoglobin concentration measured every 30 min by a blood gas analyzer.

Figure 3 shows the comparison of gasHb and BIS. GasHb was measured every 30 min, and BIS was measured continuously. A comparison of these data shows a correlation with a maximum r3 value of 0.969. We confirmed the correlation between gasHb and BIS through their observed similar fluctuations.

### Comparison of StO_2_ and BIS

**Figure 4:**
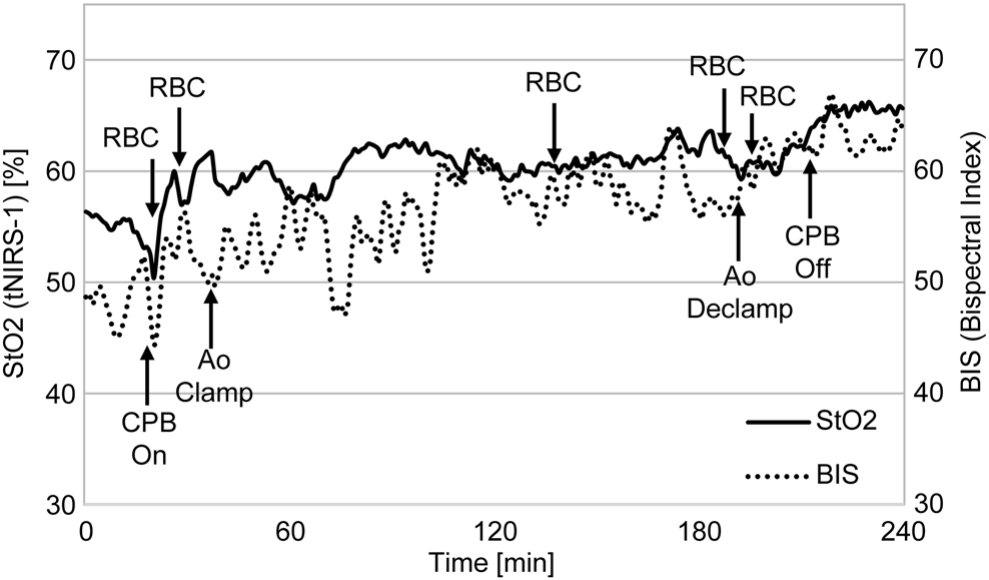
Trends of StO_2_ and BIS. This graph shows the measured changes of StO_2_ and BIS during cardiac surgery, for each event.

In the comparison of StO_2_ and BIS, during the operation (Fig. 4) it was observed that the fluctuation in StO_2_ was similar to that in BIS. In particular, it was confirmed that the fluctuations decrease at the beginning of cardiopulmonary bypass and increase after aortic cross clamping and declamping. At the time of blood transfusion, a few BIS values decreased, while almost all the StO_2_ and BIS values tended to increase. In addition, it was considered that the increase in cerebral tissue blood saturation corresponded with that of hemoglobin concentration.

### Fluctuation of estHb and gasHb

**Figure 5:**
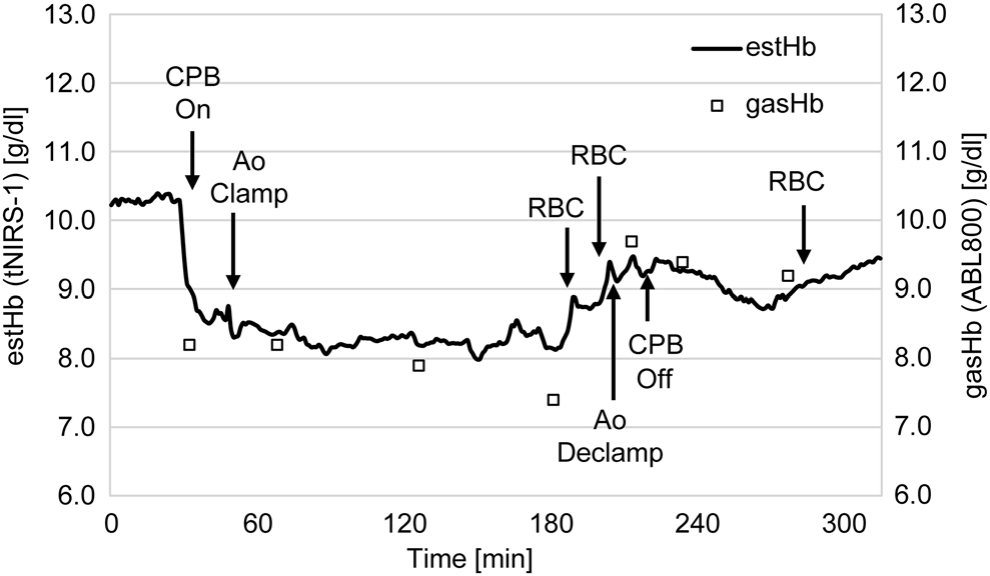
Trends of estHb and gasHb. The graph compares the change of the estimated hemoglobin concentration with hemoglobin concentration during cardiac surgery.

Figure 5 shows the fluctuation of the estHb and gasHb during the operation. EstHb level was correlated with gasHb during the operation from the beginning of cardiopulmonary bypass. Moreover, the estHb continuously captured the increase in hemoglobin concentration after blood transfusion. As the measurement error of gasHb was within 10%, this method was considered to be effective as a measurement method during operation.

### Fluctuation of BIS and gasHb

**Figure 6:**
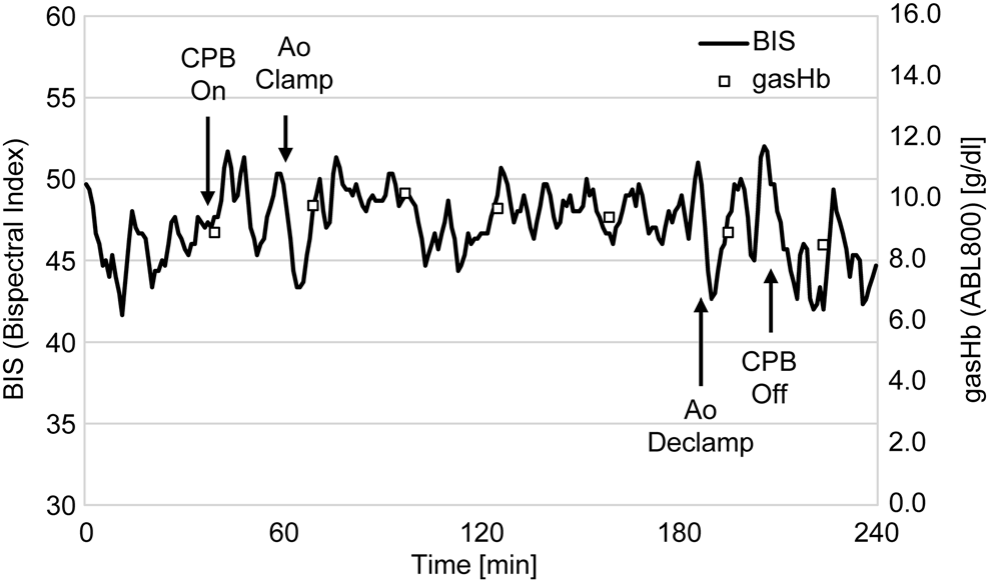
Trends of BIS and gasHb. The graph compares the change of hemoglobin concentration with continuously measured BIS during cardiac surgery.

Figure 6 shows the comparison of the BIS and gasHb. BIS was measured continuously and gasHb was measured every 30 min, showing the same fluctuation observed during the operation. By comparing the fluctuation ratio at every measurement section, correlation was confirmed as the differential of all sections was within 10%. As a result, it was found that the combined use of tNIRS-1 and BIS is a noninvasive and effective monitoring method for determining the oxygen status of cerebral tissues.

## Discussion

To understand the physiological condition of the patient and appropriately control the cardiopulmonary bypass system during cardiac surgery, it is necessary to obtain rapid and accurate physiological information. In addition, it is important to measure and understand cerebral tissue oxygen saturation to prevent irreversible damage^9–13^.

In particular, owing to NIRS, a field that has seen rapid progress in recent years, it is possible to measure cerebral tissue oxygen saturation and hemoglobin concentration in newborns and adults. Meanwhile, BIS has facilitated measurement of EEG noninvasively and continuously during cardiac surgery.

These instruments have been used in various fields, such as cardiac surgery, however almost all of the measurement data collected by conventional NIRS are relative values^14,15^, requiring the sensor to be recalibrated when reattached or misaligned. In contrast, tNIRS-1 used in this study can measure absolute values of StO_2_ and estHb. When the sensor is reattached or misaligned, the effect on the measured values is minimal, and recalibration is not required.

Additionally, the brain has different sensitivities to hypoxia in each area, and it is believed that the cerebral cortex, diencephalon, midbrain, and medulla oblongata are easily damaged in this order^16,17^. Hence, we determined that the incidence rate of brain damage could be reduced by detecting hypoxia in the cerebral cortex at an early stage.

The purpose of this study was to investigate the effective use of tNIRS-1, which can measure cerebral tissue oxygen saturation and hemoglobin concentration noninvasively and continuously, and evaluate its combined use with BIS.

First, the fluctuation of StO_2_ by tNIRS-1 and BIS was measured. Only a few studies have reported the use of tNIRS-1 as a clinical instrument. However, BIS has been reported in many studies to be useful for determining index of sedation and cerebral ischemic monitor^18,19^. We therefore examined the relationship of BIS with StO_2_, which represents the oxygen saturation of brain tissue. If the correlation between tNIRS-1 and BIS can be demonstrated, it would be possible to shift to the use of only tNIRS-1 in the future.

As shown in Fig. 1, a positive correlation was observed between StO_2_ and BIS. Although the principle of BIS measurement is not publicly available, Glass et al^20^. reported that the database used to calculate BIS values includes four types of anesthetics: isoflurane, thiopental, propofol, and midazolam, in addition to nitrous oxide or narcotics. The most common type of anesthetics used in each case in this study was midazolam, and the measured BIS values were presumed to be reliable.

Therefore, we believe that we can respond flexibly to hypoxia by monitoring both StO_2_ and BIS values, or simply one of them, when the oxygen status of cerebral tissue changes.

Figure 2 shows the comparison between gasHb and estHb. There is a favorable correlation between the two parameters, but in some cases, subsequent measurements are affected by the sensing state at the start of the measurement. This implies that for the case of estHb, the value was lower (or higher) than gasHb at the start of the measurement, and the measurement remained lower (or higher) throughout the operation. It was not possible to determine the effect of this factor on the sensor-side or patient-side. However, using the initial value as a reference for gasHb and estHb, and taking the difference into consideration, it was possible to measure estHb without significant deviation until the end of the operation. Therefore, more cases need to be considered.

In Fig. 3, although gasHb was correlated with BIS, the results were scattered overall. One of the reasons for this variability is that the data output by BIS is approximately read every 60 s, whereas for gasHb it is only measured at a particular time.

BIS values were displayed on a monitor at one second intervals and compared to the intermittent measurement of gasHb, adequate for capturing the fluctuation. However, as the BIS measurements are sensitive, the impedance of each electrode must be less than a specific value to suppress the effect of noise. Furthermore, the fluctuation in the head and the detachment of the sensor position are considered to be other factors affecting the measurement.

Therefore, it is important to place the sensor on the forehead before the start of measurement, ensuring that the sensor is not detached during operation – this is a process to be examined in a future study.

The values of StO_2_ and BIS decreased at the start of the cardiopulmonary bypass system, as shown in Fig. 4. It is considered that the priming solution in the cardiopulmonary bypass system diluted the patient’s blood and captured the temporary hypoxia caused by the decreased hemoglobin concentration in the blood.

Although there were differences in the fluctuation ratio after aortic cross clamping and declamping, both showed an upward trend. This trend may capture the process of improving hypoxia in cerebral tissue as temporary clamped blood flow is reperfused by the cardiopulmonary bypass system. After transfusion, both StO_2_ and BIS showed an increasing trend; however, some cases showed a decreasing trend. The reason for this decrease is that the physiological metabolic rate increases during the rewarming time of patient’s temperature before the end of the cardiopulmonary bypass, and the oxygen supply is insufficient to meet the oxygen demand of the patient’s body^21–23^.

Figure 5 shows the fluctuations in hemoglobin concentration, measured by tNIRS-1 and ABL800. In our hospital, the hemoglobin concentration was measured by ABL800 intermittently, and it took a few minutes to obtain results from blood collection. It was difficult to confirm the detailed fluctuation of hemoglobin concentration during the period of measurement. However, the tNIRS-1 used in this study, which can measure the fluctuation of estimated hemoglobin concentration noninvasively, showed a high correlation with gasHb. Using tNIRS-1, it is possible to not only measure hemoglobin concentration continuously, but also measure and enable an appropriate response in the case of rapid changes in the patient’s condition.

As mentioned previously, BIS and gasHb were correlated with StO_2_ and estHb measured by tNIRS-1, while BIS and gasHb were also considered to be correlated (Fig. 6). While this is considered useful for detecting hypoxia, however, it implies that BIS can be affected by the type of anesthetic used in the surgery, and therefore should be used in combination with other instruments to increase reliability^24^.

One of the challenges of this study is the comparison of instruments and patients in a large number of cases, as tNIRS-1 is a novel instrument for clinical use. Our research was conducted at a single hospital; however, comparative case studies based on data collection and analyses at other hospitals will be required to achieve reliable results.

## Methods

### Target and measurement methods

Ten adult patients, who received cardiac surgery, agreed to participate in this study (Table 1). Patients who had one or more of the following conditions; aortic regurgitation, mitral regurgitation, tricuspid regurgitation, and coronary stenosis, who underwent thoracic aorta surgery were excluded. The BIS and tNIRS-1 sensors were placed on the patients' foreheads, and the values were monitored simultaneously to enable comparison. Each measurement was performed continuously from the time of induction of anesthesia until the end of the surgery. The BIS and StO_2_ were plotted as one point every 60 s and compared for each of the 10 cases. Furthermore, we compared the variability of the estimated hemoglobin concentration (estHb) measured by tNIRS-1 and the variability of BIS against the measured patient’s blood hemoglobin concentration (gasHb), respectively. The blood hemoglobin concentration was measured at the start of cardiopulmonary bypass and then every 30 min for the comparison of gasHb, estHb, and BIS values. The number of gasHb measurements varied depending on the time of surgery, but approximately 6 to 7 points were measured per case. In addition, the multi-linear mixed models were used for each comparison. The drugs used during the induction of anesthesia in this study were midazolam or propofol. The dosages ranged from 0.10 to 0.20 mg/kg and 4 to 7 mg/kg/h, respectively. The anesthetic used during cardiopulmonary bypass was propofol at approximately 2 mg/kg/h.

**Table 1:** Patients information.

Gender	Age	Disease history	Height (cm)	Weight (kg)
M	70	MR	162	82.2
F	83	MR, TR, af, AP	151	49.8
F	67	MR, TR, af	167	58.8
M	81	MR, TR, af	169	52.7
F	65	AR, MR, TR	149	38.2
M	67	AR, AAE	184	57.8
M	71	EAP	160	51.3
F	59	MR	153	55.7
F	82	MR, TR	144	45.7
M	82	AP	157	38.8

This table shows patients information, which are gender, age, disease history, height, and weight.

*AR* aortic valve regurgitation, *AP* angina pectoris, *MR* mitral valve regurgitation, *AAE* annulo aortic ectasia, *TR* tricuspid valve regurgitation, *EAP* effort angina pectoris, *af* atrial fibrillation.

### Ethics apporval and consent to participate

The study was approved by Showa University Northern Yokohama Hospital clinical ethics committee (ethical approval ID: 18H028) and conducted in accordance with the relevant guidelines and regulations in compliance with the Showa University Northern Yokohama Hospital clinical ethics committee. Informed consent was obtained from all study participants.

## Abbreviations

StO_2_: Tissue Oxygen Saturation
gasHb: Blood gas analyzed Hemoglobin concentration
tNIRS-1: Cerebral Tissue Oximeter using Near Infrared Time-Resolved Spectroscopy
BIS: Bispectral Index
estHb: Estimated Hemoglobin concentration
EEG: Electroencephalogram
NIRS: Near Infrared Spectroscopy
EMG: Electromyography
SRS: Spatially Resolved Spectroscopy
PRS: Phase Resolved Spectroscopy
TRS: Time Resolved Spectroscopy
O2Hb: Oxygenated Hemoglobin concentration
HHb: Deoxygenated Hemoglobin concentration
tHb: Total Hemoglobin concentration
RBC: Red Blood Cell
CPB On: Cardio Pulmonary Bypass On
CPB Off: Cardio Pulmonary Bypass Off
Ao Clamp: Aorta Clamping
Ao Declamp: Aorta Declamping
